# Supplementary material for: Small molecule modulation of the Drosophila Slo channel elucidated by cryo-EM
Source: Nat Commun. 2021 Dec 9;12:7164. doi: 10.1038/s41467-021-27435-w (PMC8660915; doi:10.1038/s41467-021-27435-w)
Supplement: Supplementary file 1 — Supplementary Information [file 41467_2021_27435_MOESM1_ESM.pdf]

Supplementary Information

Small molecule modulation of the *Drosophila* Slo channel elucidated by cryo-EM

Tobias Raisch, Andreas Brockmann, Ulrich Ebbinghaus-Kintscher, Jörg Freigang, Oliver Gutbrod, Jan Kubicek, Barbara Maertens, Oliver Hofnagel, Stefan Raunser

Supplementary figures

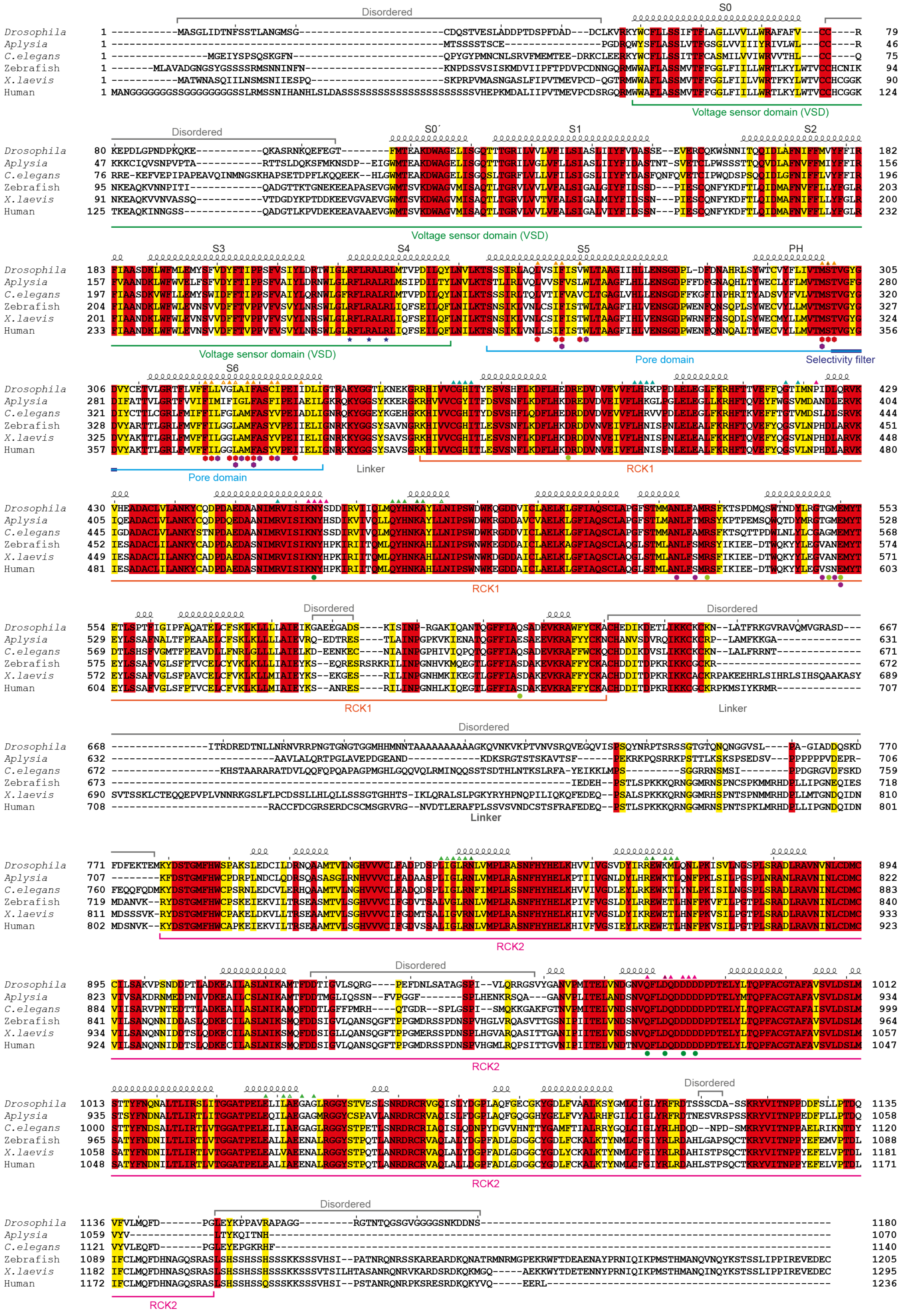

Supplementary Figure 1. Sequence alignment of Slo

Sequence alignment of metazoan Slo proteins. Amino acids which are identical between the six species are highlighted by a red background, highly conserved residues by a yellow background. Secondary structure elements as observed in our *Drosophila* Slo structure are indicated above the alignment, structural domains below the alignment. Loops and linkers which are disordered in our structures are indicated in grey. Residues involved in binding verruculogen and emodepside are highlighted by red and purple hexagons below the alignment, respectively. The gating charge residues in helix S4 are marked by blue asterisks. Amino acids involved in coordination of the Ca^{2+} and Mg^{2+} ions in the gating ring are indicated by green and purple circles, respectively. Residues lining the predicted binding pockets in *Drosophila* Slo are indicated by triangles above the alignment in the colors corresponding to Fig. 2A,B.

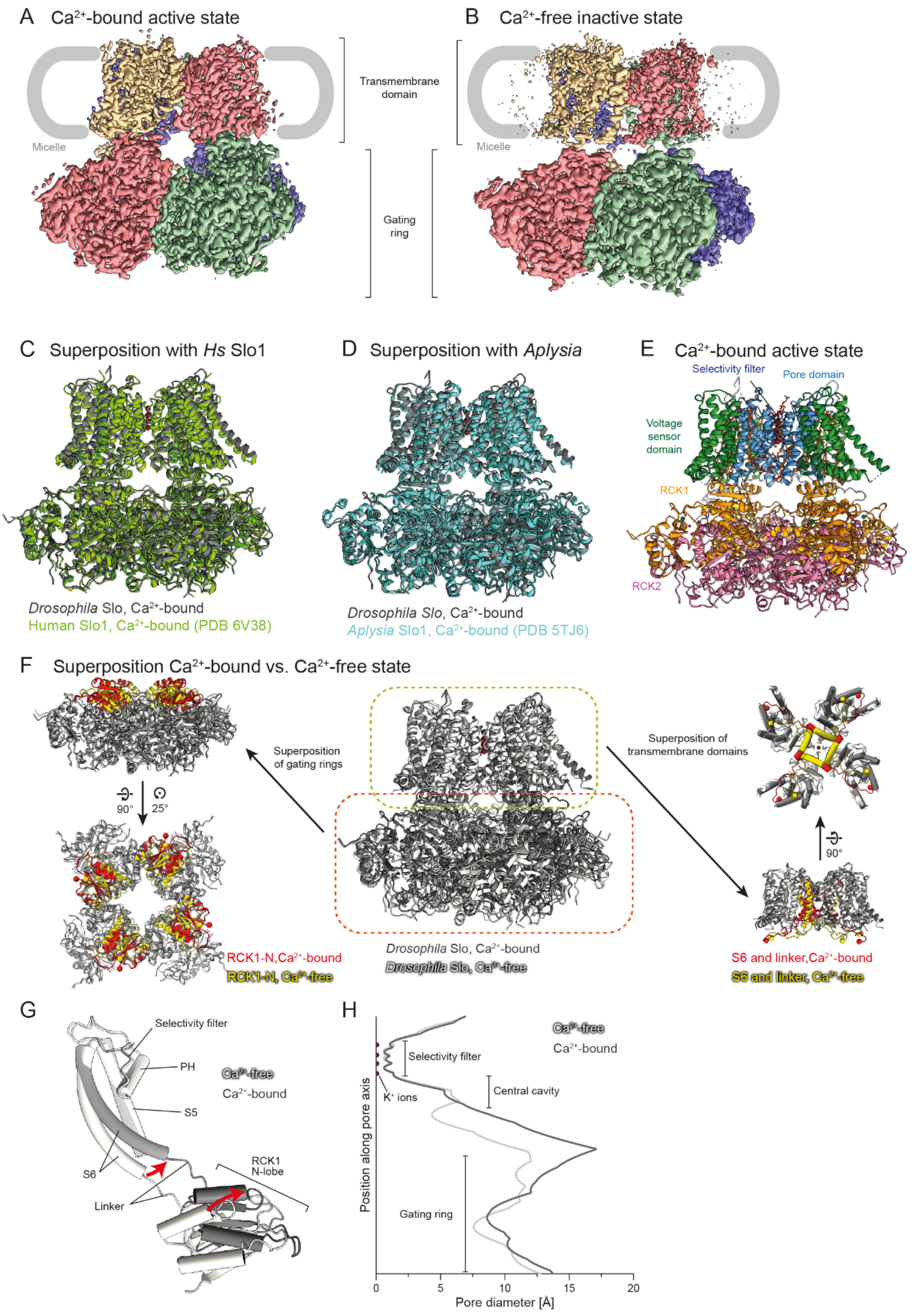

Supplementary Figure 2. Analysis of Slo apo structures

A) Cryo-EM reconstruction of Ca^{2+}-bound Slo colored by macromolecular chain as in Fig. 1C. The position of the micelle is indicated by a thick grey line.

B) Cryo-EM reconstruction of Ca^{2+}-free Slo colored by macromolecular chain as in panel A. The position of the micelle is indicated by a thick grey line.

C) Superposition of the Ca^{2+}-bound states of *Drosophila* Slo (grey; this work) and human Slo1 (green; PDB 6V38; https://doi.org/10.2210/pdb6V38/pdb).

D) Superposition of the Ca^{2+}-bound states of *Drosophila* Slo (grey; this work) and *Aplysia* Slo1 (cyan; PDB 5TJ6; https://doi.org/10.2210/pdb5TJ6/pdb).

E) Structure of Slo in the Ca^{2+}-bound active conformation shown from the side. Domains are colored according to Fig. 1A. Ions are shown as spheres (K^+: Red; Ca^{2+}: Green; Mg^{2+}: Pink). Phosphatidylcholine and cholesterol molecules are shown as sticks.

F) Superposition of the Ca^{2+}-bound (dark grey) and Ca^{2+}-free (light grey) conformations. The panel in the center shows a global superposition. For the panels on the left side, the TMDs were omitted and the gating rings superposed to visualize the Ca^{2+}-induced movement of the RCK1 N-lobe. For the panels on the right side, the gating rings were omitted and the TMDs superposed to show the conformational change of helix S6 induced by the movement of the RCK1 N-lobe.

G) Superposition of the pore domain and the RCK1 N-lobe in the Ca^{2+}-free (white) and Ca^{2+}-bound state (grey). As the RCK1 N-lobe shifts outwards upon Ca^{2+}-binding, it drags along the linker and kinks helix S6.

H) Plot showing the pore diameter of the central ion conducting channel along the central fourfold axis in the Ca^{2+}-bound active (dark grey) and the Ca^{2+}-free inactive conformation (light grey). The pore is narrowest at the selectivity filter at the height of the outer leaflet of the membrane. Large changes of pore diameter are observed below the central cavity where the conformational change of helix S6 leads to a strong expansion of pore diameter. The positions of the K^+ ions in the selectivity filter are indicated by red half-circles.

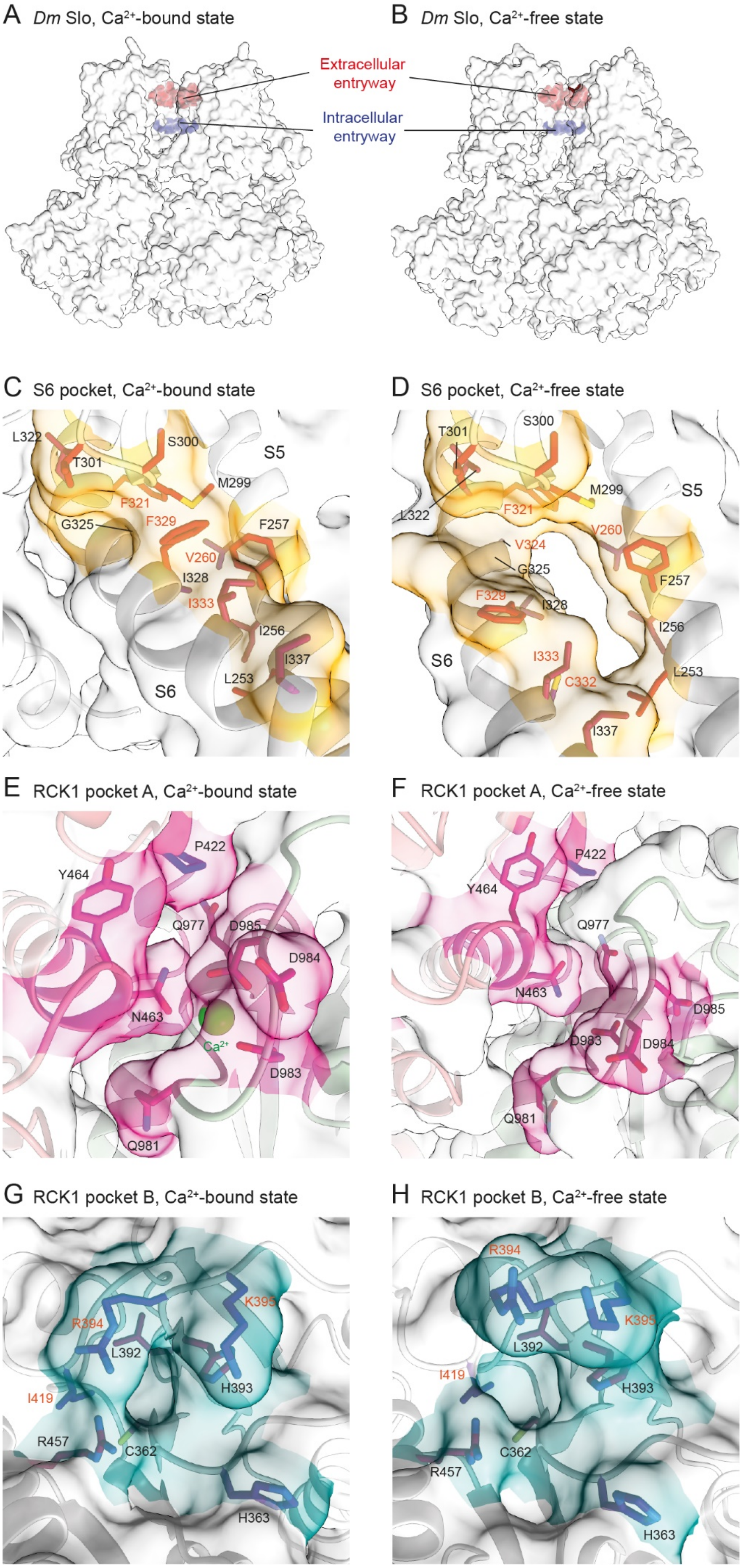
A Dm Slo, Ca2+-bound state
B Dm Slo, Ca2+-free state
Extracellular entryway
Intracellular entryway
C S6 pocket, Ca2+-bound state
L322
S300
T301
S5
M299
F321
F329
G325
F257
V260
I328
I333
I256
I337
L253
S6
D S6 pocket, Ca2+-free state
S300
T301
M299
S5
L322
F321
V324
V260
G325
F257
F329
I328
I256
S6
I333
C332
L253
I337
E RCK1 pocket A, Ca2+-bound state
P422
Y464
Q977
D985
D984
N463
Ca2+
D983
Q981
F RCK1 pocket A, Ca2+-free state
P422
Y464
Q977
N463
D985
D983
D984
Q981
G RCK1 pocket B, Ca2+-bound state
K395
R394
L392
H393
I419
R457
C362
H363
H RCK1 pocket B, Ca2+-free state
R394
K395
L392
I419
H393
C362
R457
H363

Supplementary Figure 3. Surface analysis of *Drosophila* Slo

A) Surface representation of the Ca^{2+}-bound conformation of Slo with the intracellular entryway highlighted in blue and the extracellular entryway shown in red. Targeting these entryways to the selectivity filter would allow physical block of K^{+} translocation, but the chemical nature and high conservation of these sites would make design of specific and potent small molecules very challenging.

B) Surface representation of the Ca^{2+}-free conformation of Slo with the intracellular entryway highlighted in blue and the extracellular entryway shown in red.

C) Close-up view of the S6 pocket location in the Ca^{2+}-bound active conformation. The pocket is completely closed and buried by the shifted helix S6.

D) Close-up view of the S6 pocket in the Ca^{2+}-free inactive conformation. The shape of the pocket is indicated as a yellow surface. Amino acid side chains that line the pocket are shown in stick representation and colored according to conservation from blue (low conservation) to red (high conservation). Residues which are identical between *Drosophila* Slo and human Slo1 are labelled with black labels, residues which differ with orange labels.

E) Close-up view of RCK1 pocket A in the Ca^{2+}-bound active conformation. The shape of the pocket is indicated as a pink surface. Amino acid side chains that line the pocket are shown in stick representation and colored according to conservation from blue (low conservation) to red (high conservation). The pocket coincides with the inter-subunit Ca^{2+}-binding site and is highly conserved. All involved side chains are identical between *Drosophila* Slo and human Slo1.

F) Close-up view of RCK1 pocket A in the Ca^{2+}-free inactive conformation. The shape of the pocket is very different from the Ca^{2+}-bound conformation.

G) Close-up view of RCK1 pocket B in the Ca^{2+}-bound active conformation. The shape of the pocket is indicated as a cyan surface. Amino acid side chains that line the pocket are shown in stick representation and colored according to conservation from blue (low conservation) to red (high conservation). Residues which are identical between *Drosophila* Slo and human Slo1 are labelled with black labels, residues which are not identical with orange labels.

H) Close-up view of RCK1 pocket B in the Ca^{2+}-free inactive conformation. The shape of the pocket is very different from the Ca^{2+}-bound conformation.

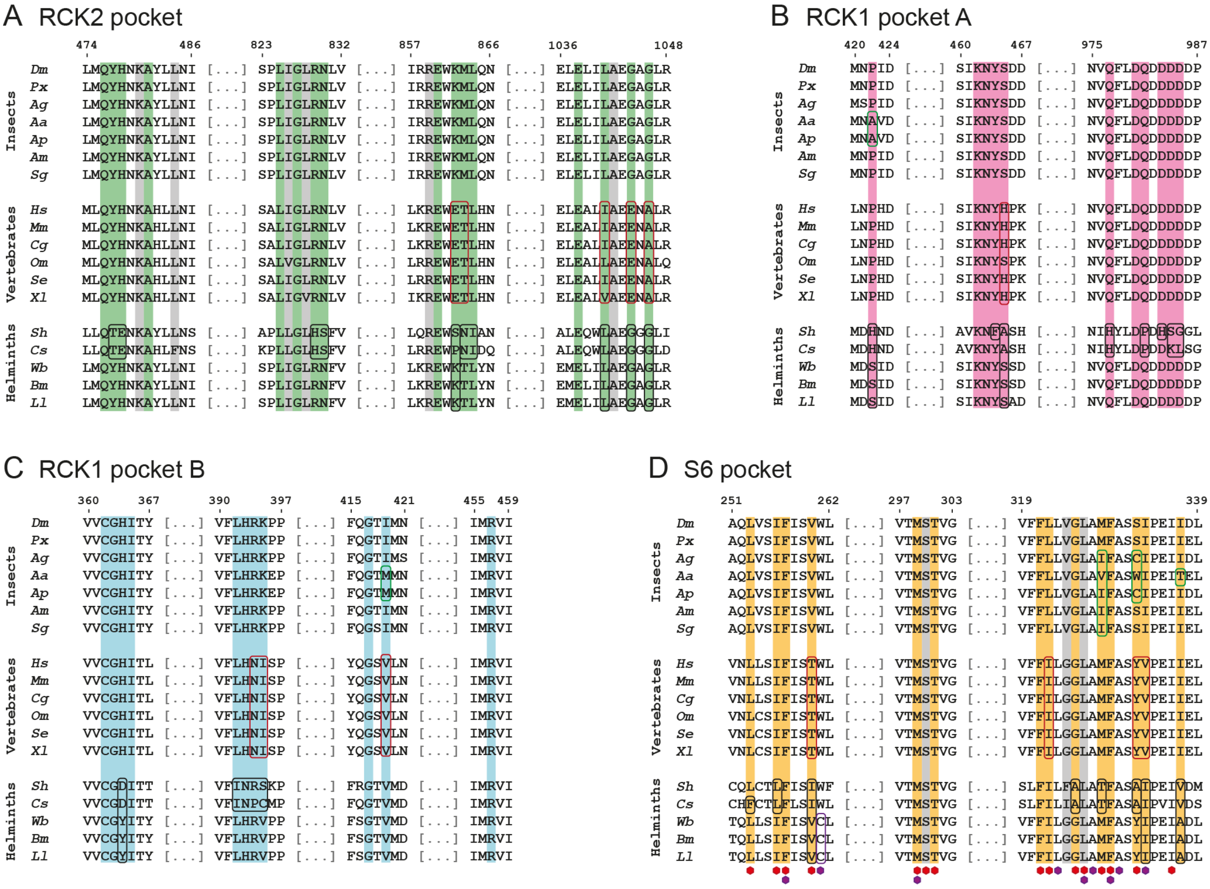

E Species and accession numbers

Insects	Dm	*Drosophila melanogaster*	Fruit fly	Uniprot Q03720
	Px	*Plutella xylostella*	Cabbage moth	Uniprot A0A7D5R4S8
	Ag	*Anopheles gambiae*	African malaria mosquito	Uniprot Q7PQJ0
	Aa	*Aedes aegypti*	Yellow fewer mosquito	Uniprot A0A6I8TSM1
	Ap	*Acyrthosiphon pisum*	Pea aphid	Uniprot J9KB00
	Am	*Apis mellifera*	Honey bee	Uniprot A0A7M7SQ65
	Sg	*Schistocerca gregaria*	Desert locust	NCBI QVD39223
Vertebrates	Hs	*Homo sapiens*	Human	Uniprot Q12791
	Mm	*Mus musculus*	Mouse	Uniprot Q08460
	Cg	*Cricetulus griseus*	Chinese hamster	Uniprot A0A061IPY6
	Om	*Oncorhynchus mykiss*	Rainbow trout	NCBI XP_036842799
	Se	*Sitta europaea*	Eurasian nuthatch	Uniprot A0A7L1UUR8
	Xl	*Xenopus laevis*	Western clawed frog	Uniprot Q90ZC7
Helminths	Sh	*Schistosoma haematobium*	Blood fluke	NCBI XP_035589316
	Cs	*Clonorchis sinensis*	Chinese liver fluke	Uniprot A0A419QD48
	Wb	*Wuchereria bancrofti*	Roundworm	Uniprot A0A3P7DPJ7
	Bm	*Brugia malayi*	Filarial nematode worm	Uniprot A0A0J9YAQ9
	Ll	*Loa loa*	Eye worm	Uniprot A0A1I7VB66

Supplementary Figure 4. Sequence alignments of potentially druggable pockets

A) - D) Sequence alignments of amino acids lining the four pockets identified by BiteNet, i.e. the RCK2 pocket (**A**), the RCK1 pocket A (**B**), the RCK1 pocket B (**C**) and the S6 pocket (**D**), including insect, vertebrate and helminth species. Residue numbers above the alignments correspond to *Drosophila* Slo. Residues which line the pockets involving their side chains are highlighted in the colors assigned to the respective pockets in Fig. 2A,B, residues which contribute only by their main chain are indicated by a grey background. Pocket-lining residues of insect species that deviate from the *Drosophila* sequence and might therefore give rise to structural differences of the pockets between these insects are marked by green boxes.

Residues which differ between vertebrate and insect species and might allow design of insect-specific Slo modulators are highlighted by red boxes. Amino acids of helminth species that deviate from mammalian Slo and could be utilized for development of novel anthelmintics are indicated by blue boxes. Red and purple hexagons below the cavity pocket alignment in panel **B** indicate residues interacting with verruculogen and emodepside, respectively, and a purple box marks deviations from the consensus sequence in several nematode species outside the cavity-lining residues which might affect emodepside binding.

E) List of species used in the alignments in panels **A**-**D**, including (from left to right) the used abbreviation, the Latin and English species names and the Uniprot or NCBI accession numbers.

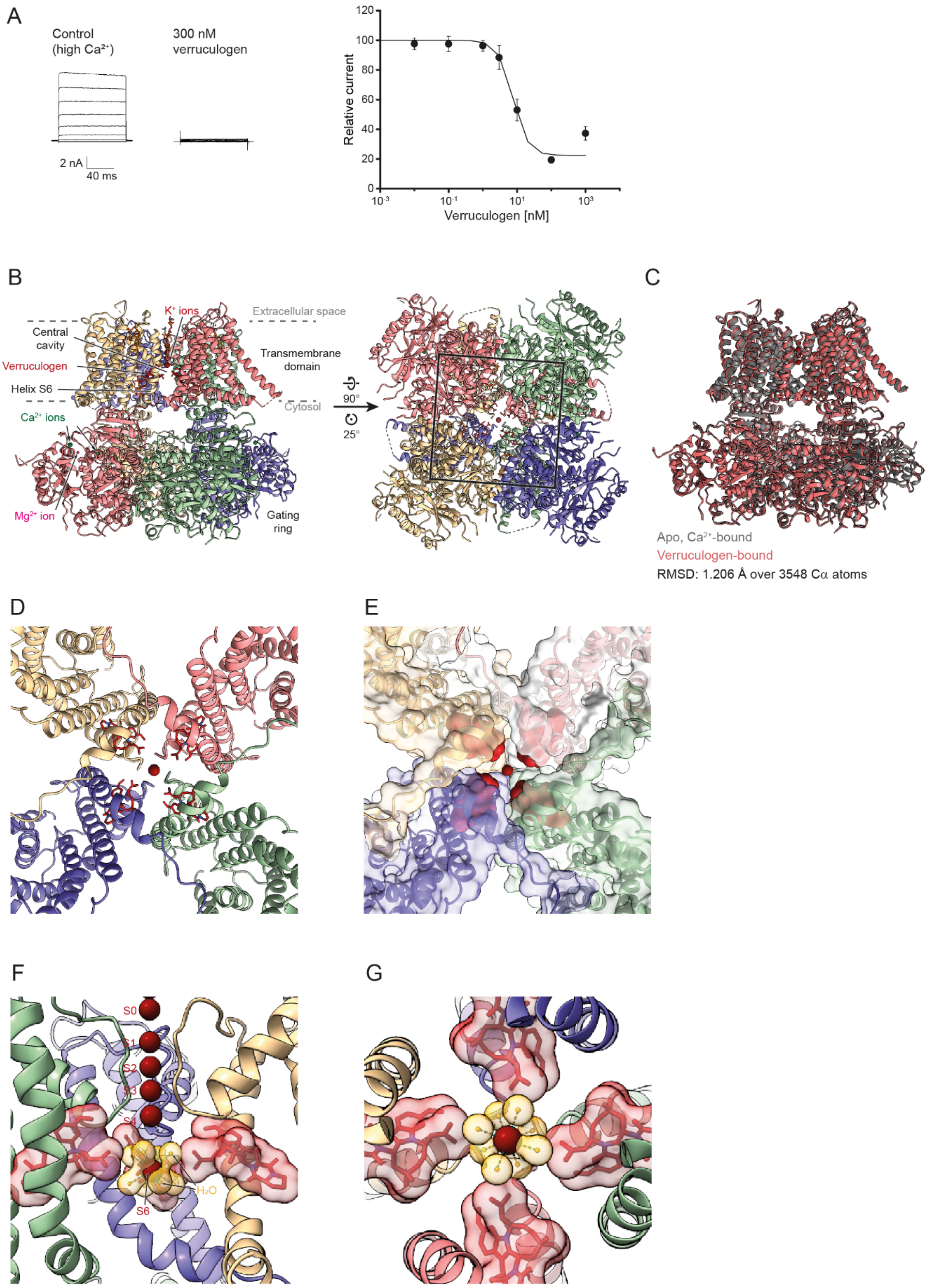

Supplementary Figure 5. Structure of verruculogen-bound Slo

A) Inhibition of Slo conductance by verruculogen. The left panels show representative current traces of CHO cells stably expressing Slo in presence and absence of 300 nM verruculogen under high Ca^{2+} conditions. Traces are shown for subsequent 100 ms measurements at membrane potentials between -120 and +60mV in 10mV increments. The right panel shows the

dose-response curve for verruculogen on Slo. Data points represent the mean ± s.e. of n = 8-13 cells (selected automatically by the PatchControl384PE software; exact numbers and values are provided in the Source Data File).

B) Cartoon representation of verruculogen-bound Slo in two orientations with colors assigned by macromolecular chain. Verruculogen is displayed as red sticks.

C) Superposition of the Ca^{2+}-bound apo structure (grey) and the verruculogen-bound structure of *Drosophila* Slo (salmon).

D) View from the gating ring (omitted for unobstructed view) into the central cavity and onto the selectivity filter illustrating how the aliphatic tail of verruculogen reaches into the cavity.

E) The same view with semi-transparent surfaces of the four Slo monomers shown, as well as verruculogen as solid red surfaces illustrating the constriction of the central cavity entry by the S6 helices and by verruculogen.

F) View of the verruculogen-bound cavity shown from the side, analogously to Fig. 4B. One of the four macromolecular chains was omitted to allow an unobstructed view into the internal pore. The three remaining protein chains are shown in cartoon representation. Verruculogen is shown as sticks and semi-transparent surface. The four K^+ ions in the selectivity filter are displayed as red spheres. A hydrated K^+ ion was modelled in the S6 position by superposing a KscA structure determined under high-potassium conditions (PDB 1K4C; https://doi.org/10.2210/pdb1K4C/pdb; Zhou et al., 2001) and is shown as spheres and semi-transparent surface.

G) The same structural model including superposed hydrated K^+ as in panel **F** viewed from the gating ring, showing that the constriction of pore diameter by verruculogen would make K^+ reaching the selectivity filter unlikely.

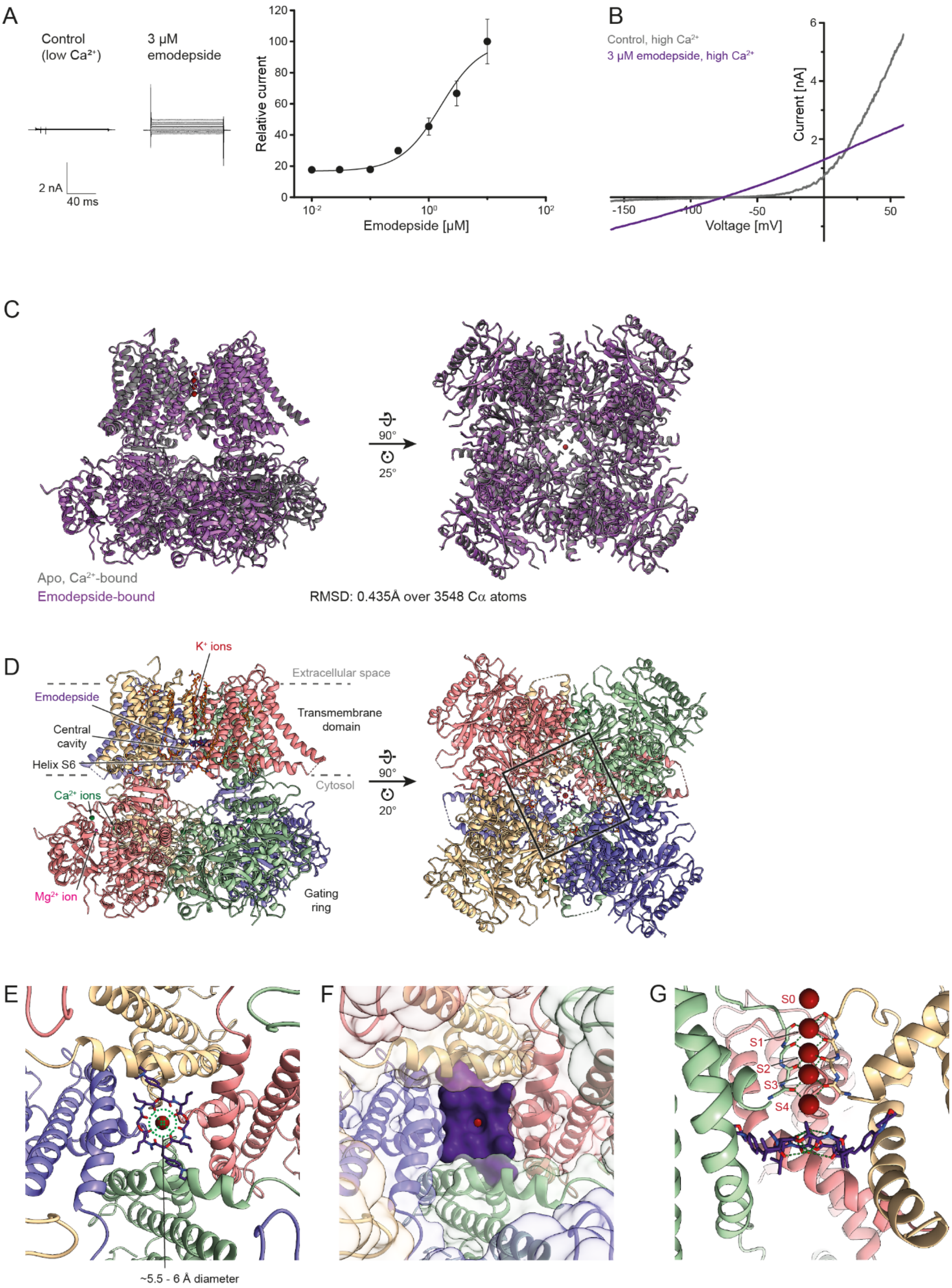

Supplementary Figure 6. Structure of emodepside-bound Slo

A) Activation of Slo conductance by emodepside at low Ca^{2+} concentration. The left panels show representative current traces of CHO cells stably expressing Slo before and after application of 3 μM emodepside under low Ca^{2+} conditions. Traces are shown for subsequent 100 ms

measurements at membrane potentials between -120 and +60mV in 10mV increments. The right panel shows the dose-response curve for emodepside on Slo. Data points represent the mean ± s.e. of n = 12-16 cells (selected automatically by the PatchControl384PE software; exact numbers and values are provided in the Source Data File).

B) Current-voltage relationship of Slo in absence and presence of emodepside under high free intracellular Ca^{2+} concentration. Whole cell currents were elicited by applying voltage ramps from -160 mV to 100 mV within 170 ms. The holding potential was clamped to -100 mV.

C) Superposition of the Ca^{2+}-bound apo structure (grey) and the emodepside-bound structure of *Drosophila* Slo (purple).

D) Cartoon representation of emodepside-bound Slo in two orientations with colors assigned by macromolecular chain. Emodepside is displayed as purple sticks.

E) View from the gating ring (omitted for unobstructed view) into the central cavity and onto the selectivity filter illustrating how emodepside serves as an additional ring limiting the access to the selectivity filter. The distances of opposite carbonyls in emodepside of ~5.5-6 Å is slightly larger compared to the carbonyls in the selectivity filter (~4.5 Å) and might pose an explanation for the reduced conductivity of emodepside-bound Slo compared to the apo situation under high Ca^{2+} conditions.

F) The same view with semi-transparent surface of the four Slo monomers shown, as well as emodepside as solid purple surfaces illustrating how emodepside constricts the pore diameter directly above the selectivity filter.

G) View of the emodepside-bound Slo central cavity shown from the side. One of the four macromolecular chains was omitted to allow an unobstructed view into the internal pore. The three remaining protein chains are shown in cartoon representation. Emodepside is shown as sticks. The four K^+ ions in the selectivity filter and the two water molecules above and below emodepside are displayed as red spheres. Hydrogen bonds between the K^+ ions and the carbonyl oxygens of the selectivity filters of two of the four subunits, as well as hydrogen bonds between the carbonyl oxygens of emodepside and the water molecules are shown as dashed lines.

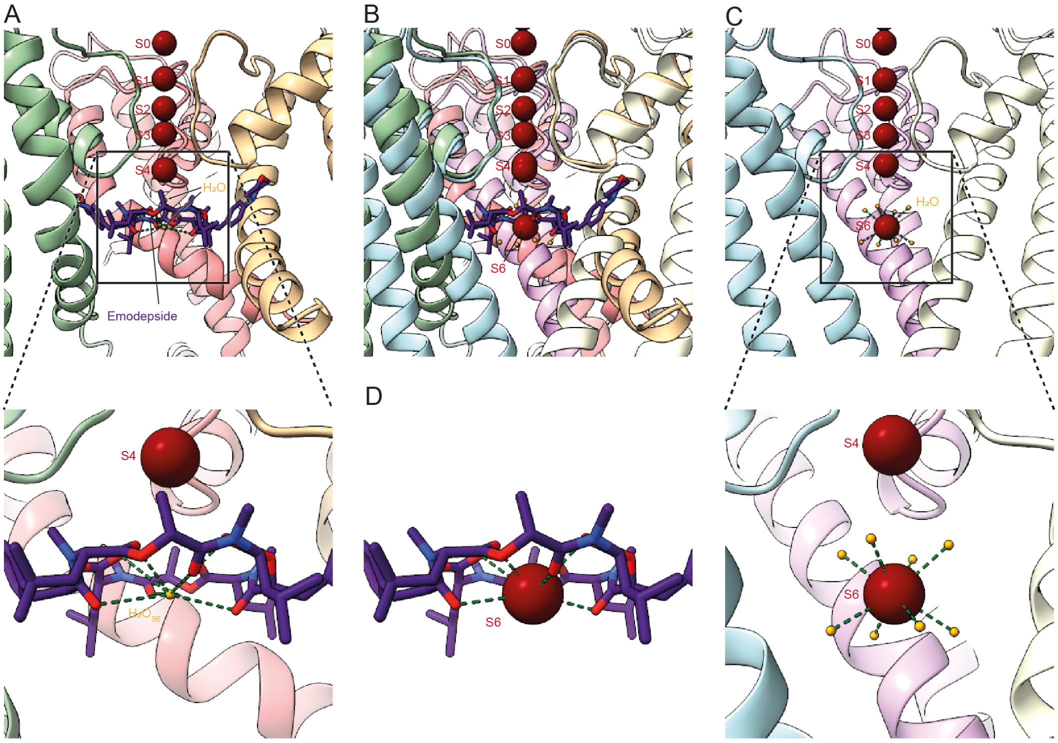

Supplementary Figure 7. Emodepside is located perfectly to stabilize K^+ in the cavity

A) View of the emodepside-bound cavity shown from the side, analogously to Fig. 5B. One of the four macromolecular chains was omitted to allow an unobstructed view into the internal pore. The three remaining protein chains are shown in cartoon representation. Emodepside is shown as sticks. The four K^+ ions in the selectivity filter are displayed as red spheres. A water molecule (yellow sphere) was modelled into a weak density in a central position slightly beneath the emodepside ring. This water is in a position where it could be weakly coordinated by the eight carbonyl oxygens of emodepside (green dashed lines).

B) Same representation as in panel **A** including a superposed crystal structure of the bacterial potassium channel KscA under high-potassium conditions (PDB 1K4C; https://doi.org/10.2210/pdb1K4C/pdb; Zhou et al., 2001).

C) Same representation as in panels **A** and **B**, but only KscA is shown. In addition to the four K^+ ions in the selectivity filter and one in the position S0 on the exterior side, another K^+ in the S6 position inside the central cavity is fully hydrated by eight water molecules.

D) Superposition of emodepside bound to Slo with the K^+ in the S6 position of KscA. The geometry of emodepside would allow to coordinate the K^+ ion in a similar but slightly distorted manner compared to a hydration shell.

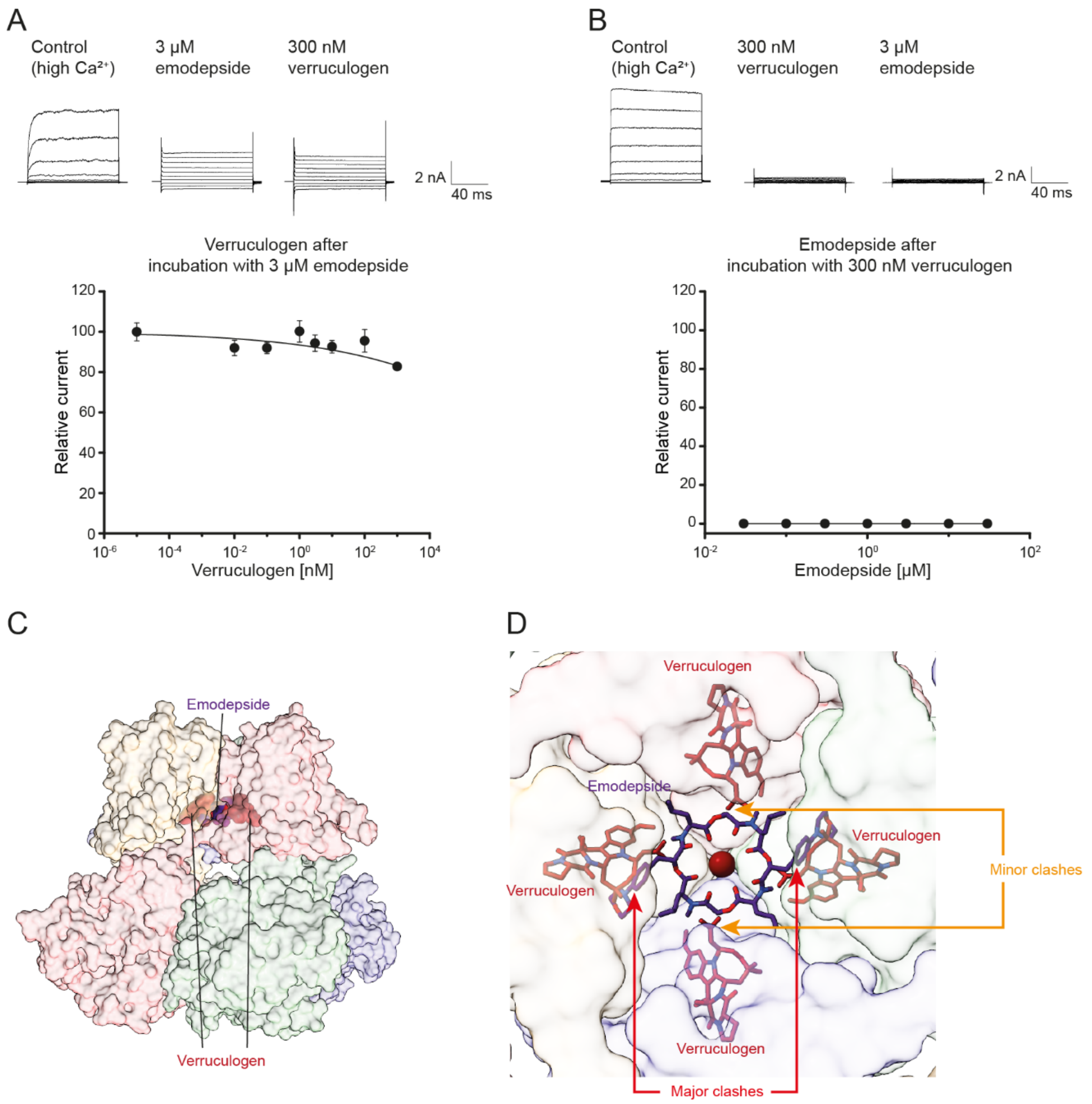

Supplementary Figure 8. Emodepside and verruculogen compete for Slo-binding

A) Emodepside-bound Slo is insensitive to verruculogen inhibition. The top panels show representative whole cell currents of CHO cells expressing Slo before (left) and after exposure to 3 µM emodepside (middle), and after subsequent exposure to 300 nM verruculogen (right). Traces are shown for subsequent 100 ms measurements at membrane potentials between -120 and +60mV in 10mV increments at a holding potential of -100mV. The lower panel shows the dose-response curve for verruculogen on Slo after application of 3 µM emodepside. Data points represent the mean ± s.e. of n = 7-11 cells (selected automatically by the PatchControl384PE software; exact numbers and values are provided in the Source Data File).

B) Emodepside cannot activate verruculogen-bound Slo. The top panels show representative whole cell currents before (left) and after exposure to 300 nM verruculogen (middle), and after subsequent exposure to 3 µM emodepside (right). Traces are shown for subsequent 100 ms measurements at membrane potentials between -120 and +60mV in 10mV increments at a

holding potential of -100mV. The lower panel shows the dose-response curve for emodepside on Slo after application of 300 nM verruculogen. Data points represent the mean ± s.e. of n = 9-13 cells (selected automatically by the PatchControl384PE software; exact numbers and values are provided in the Source Data File).

C) Verruculogen and emodepside bind to the same region in the central cavity close to the selectivity filter. The molecular model of Slo1 in the emodepside-bound conformation is shown from the side as a semi-transparent surface. The emodepside molecule is shown as a solid purple surface, the four superposed verruculogen molecules as red surfaces.

D) View from the gating ring (omitted for unobstructed view) into the central cavity and onto the selectivity filter reveals clashes between emodepside and verruculogen. Slo in the emodepside-bound conformation is shown as a semi-transparent surface, the superposed Slo in the verruculogen-bound conformation omitted for clarity. Both compounds are shown in stick representation. Simultaneous binding of both compounds is precluded as the two morpholinylphenyl groups of emodepside would severely clash with verruculogen.

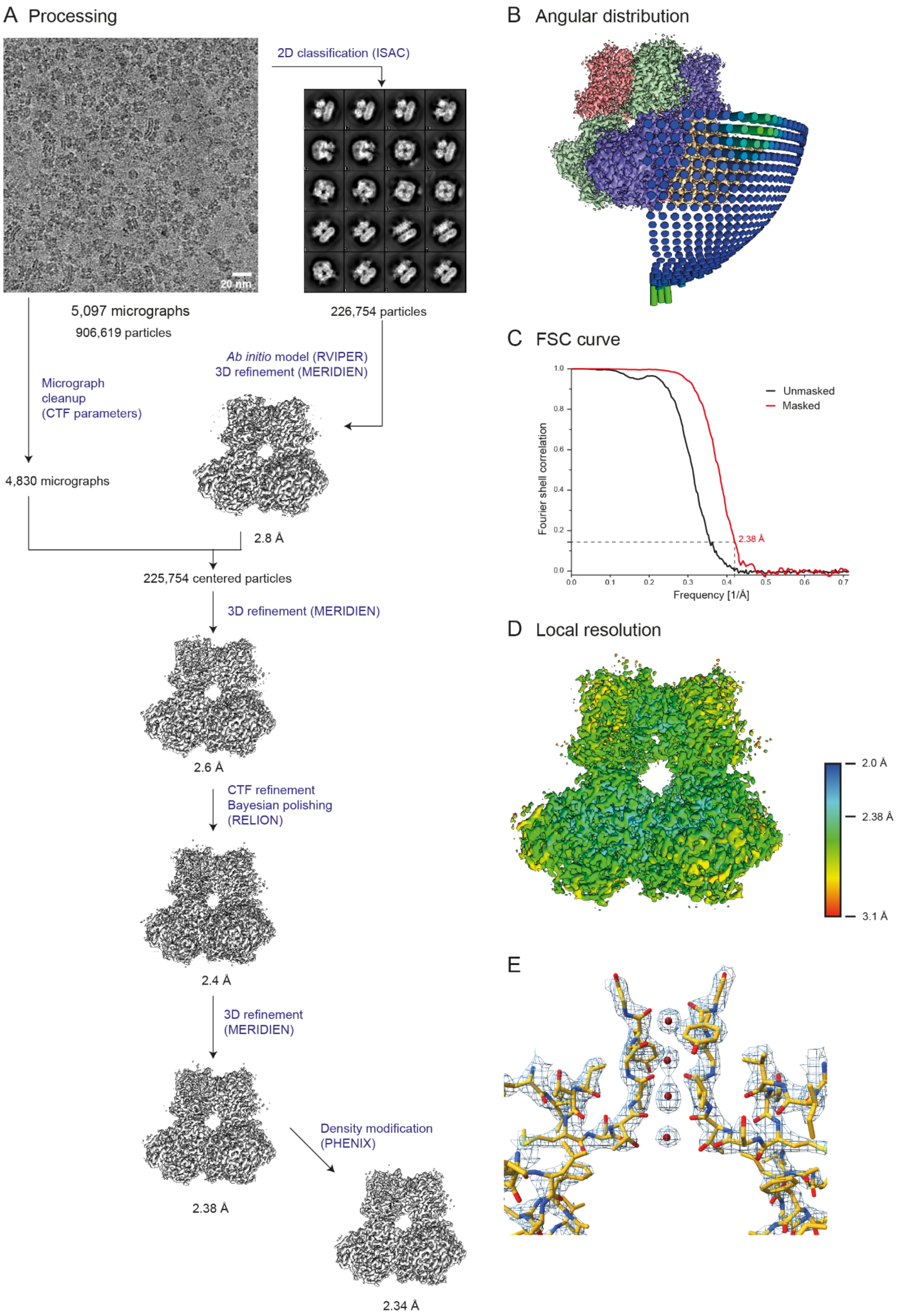

Supplementary Figure 9. Processing of Ca^{2+}-bound Slo apo EM data

A) Processing scheme including exemplary micrograph and subset of selected 2D classes of Ca^{2+}-bound *Drosophila* Slo.
B) Final reconstruction of Ca^{2+}-bound Slo. The colored bars represent angular distribution.
C) Fourier Shell Correlation (FSC) curve between two independent half-maps reconstructed from the final set of particles. The dashed line indicates the 0.143 FSC criterion that intersects the masked FSC curve at 2.38 Å.
D) Local resolution plotted on the final reconstruction in a rainbow-colored gradient from blue (2.0 Å) to red (3.1 Å).
E) Experimental Coulomb potential map in the region around the selectivity filter and central cavity. For clarity, only two of the macromolecular chains are shown.

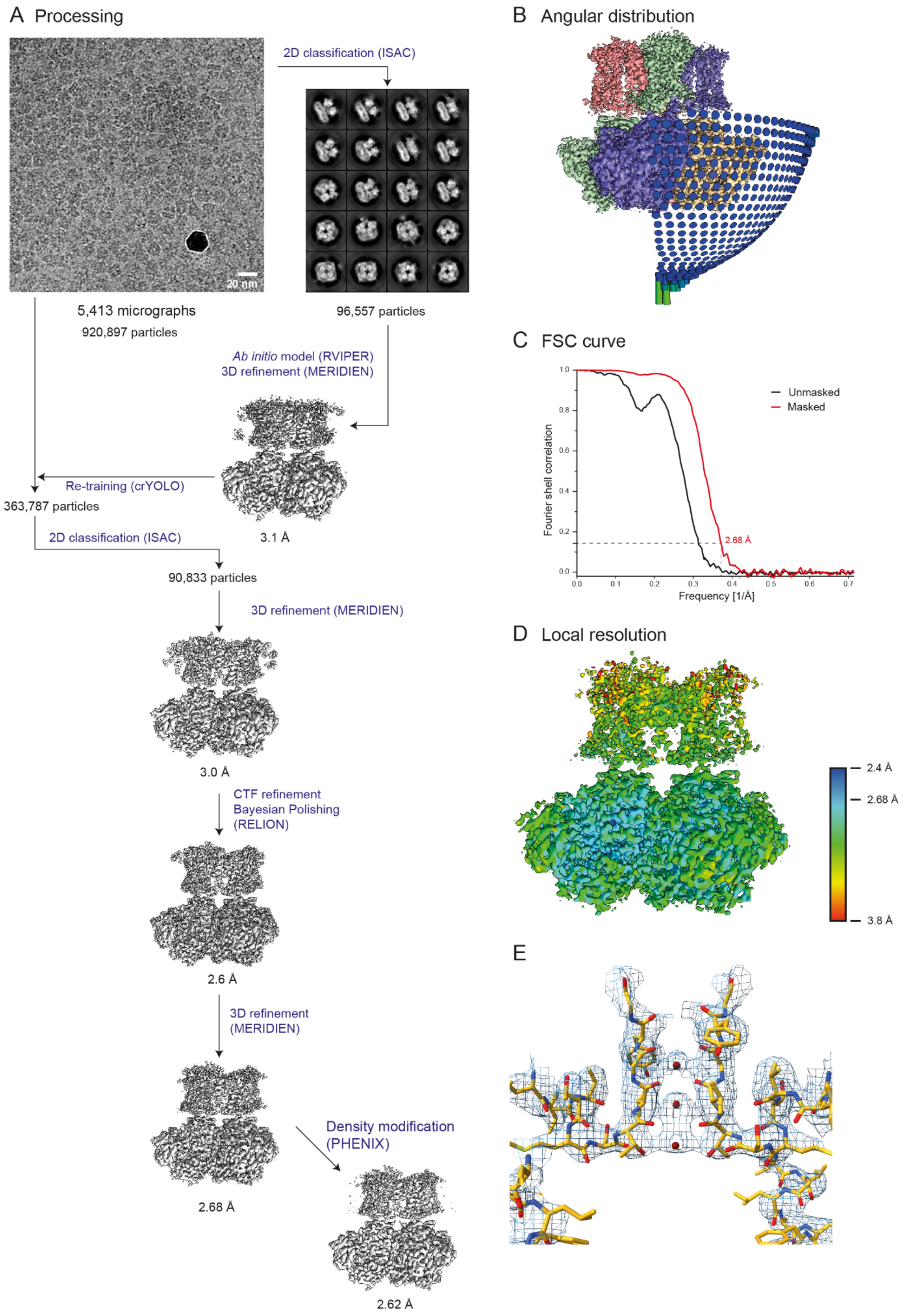

Supplementary Figure 10. Processing of Ca^{2+}-free Slo apo EM data

A) Processing scheme including exemplary micrograph and subset of selected 2D classes of Ca^{2+}-free *Drosophila* Slo.
B) Final reconstruction of Ca^{2+}-free Slo. The colored bars represent angular distribution.
C) Fourier Shell Correlation (FSC) curve between two independent half-maps reconstructed from the final set of particles. The dashed line indicates the 0.143 FSC criterion that intersects the masked FSC curve at 2.68 Å.
D) Local resolution plotted on the final reconstruction in a rainbow-colored gradient from blue (2.4 Å) to red (3.8 Å).
E) Experimental Coulomb potential map in the region around the selectivity filter and central cavity. For clarity, only two of the macromolecular chains are shown.

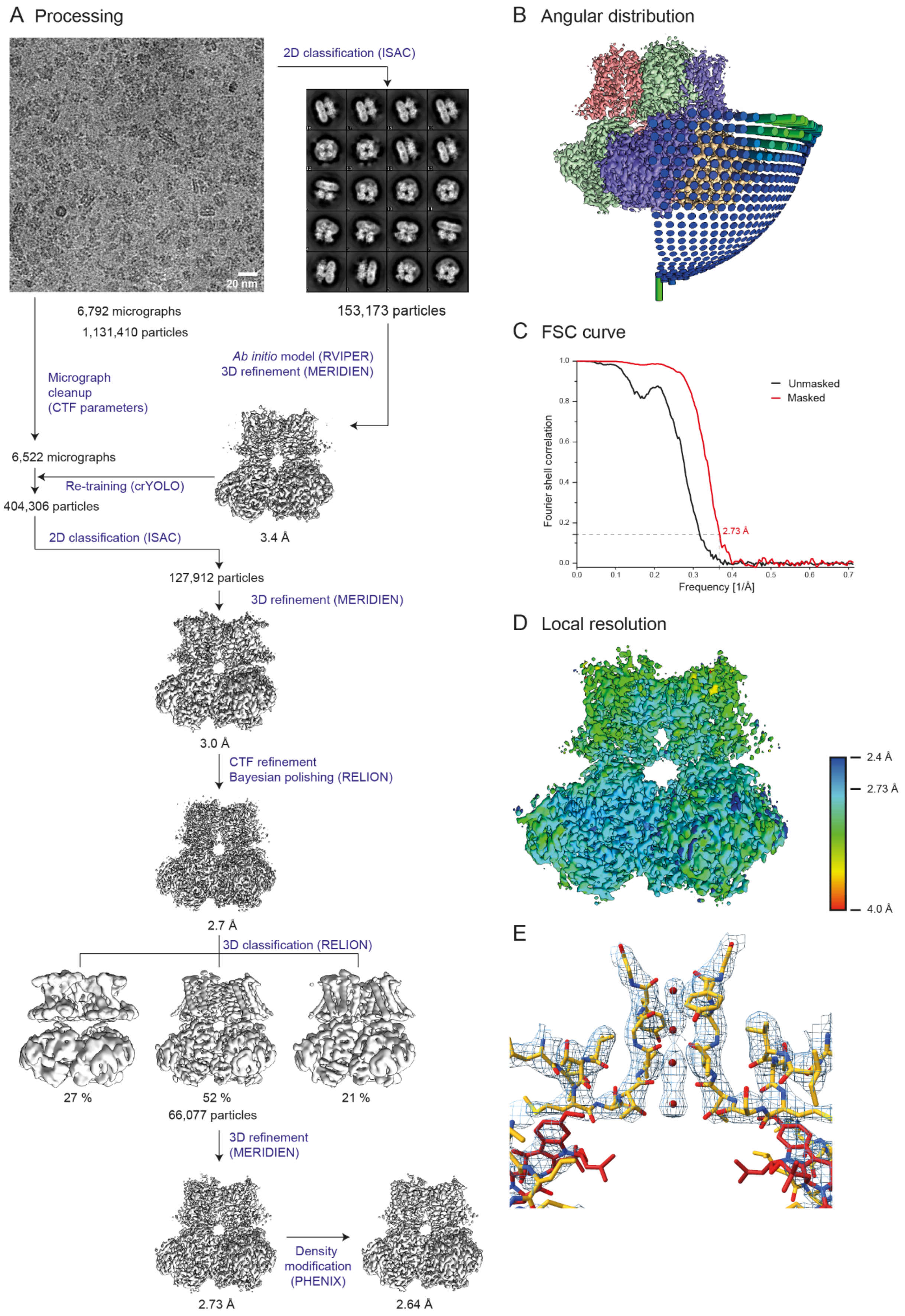

Supplementary Figure 11. Processing of verruculogen-bound Slo EM data

A) Processing scheme including exemplary micrograph and subset of selected 2D classes of verruculogen-bound Slo.
B) Final reconstruction of verruculogen-bound Slo. The colored bars represent angular distribution.
C) Fourier Shell Correlation (FSC) curve between two independent half-maps reconstructed from the final set of particles. The dashed line indicates the 0.143 FSC criterion that intersects the masked FSC curve at 2.73 Å.
D) Local resolution plotted on the final reconstruction in a rainbow-colored gradient from blue (2.4 Å) to red (4.0 Å).
E) Experimental Coulomb potential map in the region around the selectivity filter and central cavity. For clarity, only two of the macromolecular chains are shown.

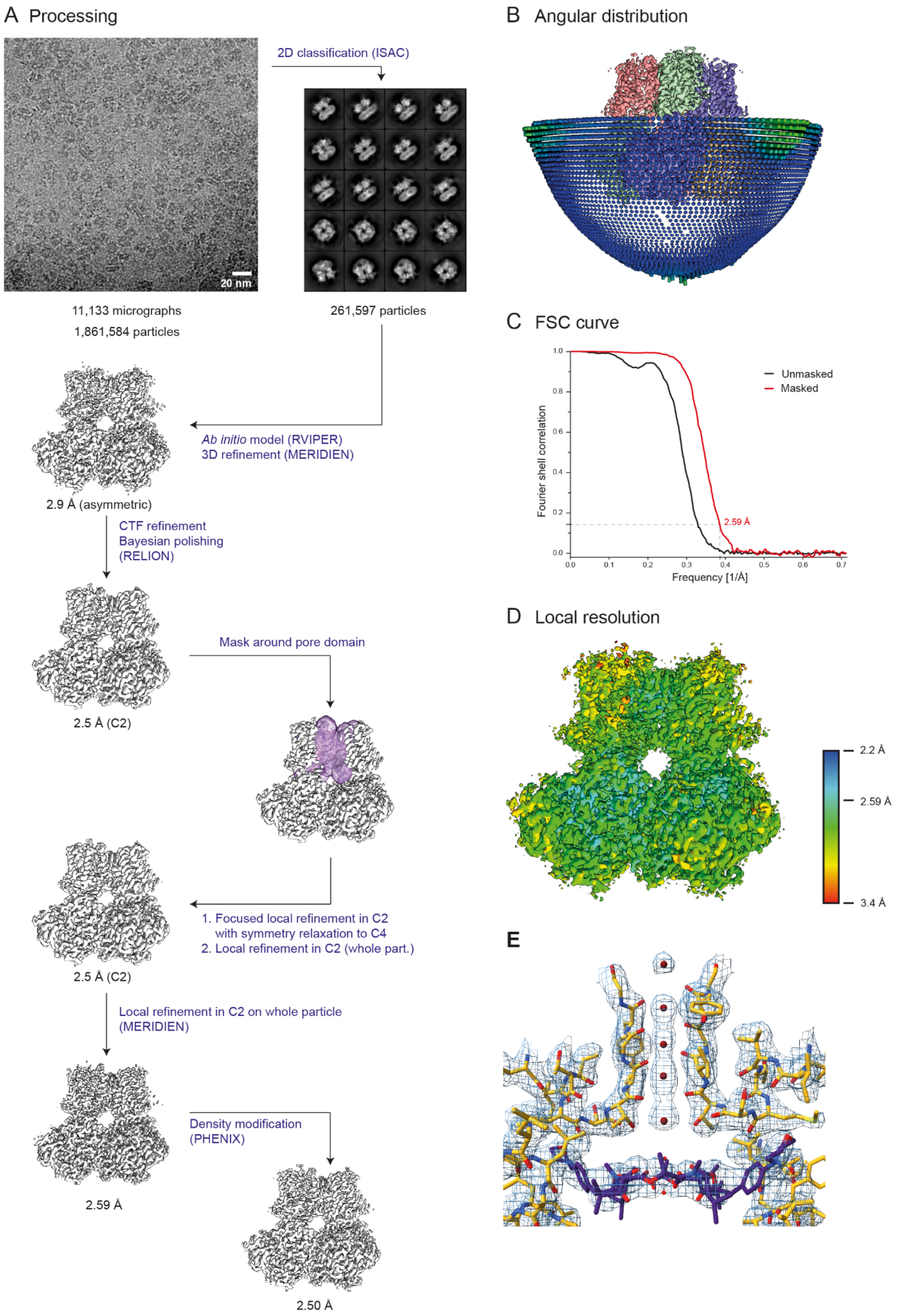

Supplementary Figure 12. Processing of emodepside-bound Slo EM data

A) Processing scheme including exemplary micrograph and subset of selected 2D classes of emodepside-bound Slo.
B) Final reconstruction of emodepside-bound Slo. The colored bars represent angular distribution.
C) Fourier Shell Correlation (FSC) curve between two independent half-maps reconstructed from the final set of particles. The dashed line indicates the 0.143 FSC criterion that intersects the masked FSC curve at 2.59 Å.
D) Local resolution plotted on the final reconstruction in a rainbow-colored gradient from blue (2.2 Å) to red (3.4 Å).
E) Experimental Coulomb potential map in the region around the selectivity filter and central cavity. For clarity, only two of the macromolecular chains are shown.

Supplementary Table 1. DNA sequence of the codon-optimized Slo gene

```
ATGGCTTCCGGCCTGATCGACACCAACTTCTCTTCTACCCTGGCTAACGGCATGTCCGGTTGCGACCAGTCCACCGTGGA
ATCTTTGGCTGACGACCCTACCGACTCTCCCTTCGACGCTGACGACTGCCTGAAAGTGCGCAAGTACTGGTGCTTCCTGC
TGTCCTCTATCTTCACCTTCCTGGCTGGCCTGCTGGTGGTGTTGCTTTGGAGGGCTTTCGCTTTCGTGTGCTGCCGTAAA
GAGCCCGACCTGGGTCCTAACGACCCCAAGCAGAAAGAGCAGAAGGCTTCCCGTAACAAGCAAGAGTTCGAGGGCACCTT
CATGACCGAGGCTAAGGACTGGGCTGGCGAGCTGATCTCTGGTCAAACCACCACCGGTCGTATCCTGGTGGTGCTGGTGT
TCATCCTGTCTATCGCTTCCCTGATCATCTACTTCGTGGACGCTTCCTCCGAAGAGGTCGAGCGTTGCCAGAAGTGGTCC
AACAACATCACCCAGCAGATCGACCTGGCCTTCAACATCTTCTTCATGGTGTACTTTTTCATCCGCTTCATTGCCGCCTC
CGACAAGCTGTGGTTTATGCTCGAGATGTACTCCTTCGTCGACTACTTCACTATCCCTCCATCCTTCGTGTCCATCTACC
TGGACCGTACCTGGATCGGCCTGCGTTTCCTTCGTGCTCTGCGTCTGATGACCGTGCCTGACATCCTGCAGTACCTGAAC
GTGCTCAAGACCTCCTCCTCCATCCGTCTGGCTCAGCTGGTTTCCATCTTCATCTCCGTGTGGCTGACCGCTGCTGGTAT
CATCCACCTGTTGGAGAACTCTGGCGACCCTCTGGACTTCGACAACGCTCACCGTCTGTCCTACTGGACCTGCGTGTACT
TCCTGATCGTGACCATGTCTACCGTCGGTTACGGCGACGTGTACTGCGAGACTGTGCTGGGTCGTACTTTCCTCGTGTTC
TTCTTGCTCGTCGGCCTGGCTATCTTCGCTTCTTGCATCCCCGAGATCATCGACCTGATCGGCACCCGTGCTAAGTACGG
TGGCACCCTGAAGAACGAGAAGGGTCGTCGTCACATCGTCGTGTGCGGTCACATCACCTACGAGTCCGTGTCTCACTTCC
TGAAGGATTTCTTGCACGAGGACCGCGAGGACGTGGACGTGGAAGTTGTGTTCTTGCACCGCAAGCCTCCAGACCTGGAA
CTCGAGGGACTGTTCAAGCGCCACTTCACCACCGTCGAGTTCTTCCAGGGAACCATCATGAACCCTATCGACCTGCAGCG
TGTGAAGGTGCACGAAGCTGACGCTTGCCTGGTGCTGGCTAACAAGTACTGTCAGGACCCCGACGCTGAGGACGCTGCTA
ACATCATGCGTGTGATCTCCATCAAGAACTACTCCGACGACATCCGCGTGATCATCCAGCTGATGCAGTACCACAACAAG
GCCTACCTGCTGAACATCCCATCCTGGGACTGGAAGCAAGGCGACGACGTGATCTGCCTGGCTGAGCTGAAGCTGGGTTT
CATTGCTCAGTCTTGCCTGGCTCCTGGTTTCTCCACCATGATGGCTAACCTGTTCGCTATGCGCTCCTTCAAGACTTCCC
CTGACATGCAGTCCTGGACCAACGACTACCTGCGTGGCACCGGCATGGAAATGTACACCGAGACTCTGTCCCCAACCTTC
ATCGGTATCCCTTTCGCTCAGGCTACCGAGCTGTGCTTCTCCAAGCTCAAGCTGCTGCTGCTCGCTATCGAGATCAAGGG
TGCTGAGGAAGGTGCTGACTCCAAGATCTCTATCAACCCTCGTGGTGCTAAGATCCAGGCTAACACCCAGGGATTCTTTA
TCGCTCAGTCCGCTGACGAAGTGAAGCGCGCCTGGTTCTACTGCAAGGCTTGCCACGAGGATATCAAGGACGAAACCCTG
ATCAAGAAATGCAAGTGCAAAAACCTGGCCACCTTCCGCAAGGGTGTCCGTGCTGTTCAAATGGTCGGACGCGCTTCTGA
CATCACCCGTGACAGAGAGGACACCAACCTGCTCAACCGTAACGTGCGTCGTCCCAACGGTACTGGAAACGGCACTGGTG
GCATGCACCACATGAACAACACAGCTGCTGCCGCTGCAGCTGCGGCTGCTGCGGGAAAGCAAGTGAACAAAGTGAAGCCC
ACCGTGAACGTGTCCCGTCAGGTTGAGGGTCAAGTGATCTCCCCTAGCCAGTACAACCGTCCTACCTCTCGTTCCTCCGG
TACTGGCACCCAAAACCAGAACGGCGGTGTCTCTCTGCCCGCTGGTATCGCTGACGATCAGTCCAAGGACTTCGATTTCG
AAAAGACCGAGATGAAGTACGACTCGACCGGCATGTTCCACTGGTCCCCAGCTAAGTCTCTCGAGGATTGCATCTTGGAC
CGTAACCAGGCTGCTATGACCGTGTTGAACGGTCACGTGGTCGTGTGCCTCTTCGCTGACCCTGACTCTCCTCTGATCGG
ACTGCGTAACCTGGTCATGCCTCTGCGCGCTTCTAACTTCCACTACCACGAACTGAAGCACGTGGTCATCGTGGGTTCCG
TGGACTACATCCGTCGCGAGTGGAAGATGCTGCAGAACCTGCCTAAGATCTCCGTGCTGAACGGCTCCCCTCTGTCTCGT
GCTGATCTGCGTGCTGTGAACGTGAACCTGTGCGACATGTGCTGCATCCTGTCCGCTAAGGTGCCATCCAACGACGATCC
CACTCTGGCTGACAAAGAGGCTATCCTGGCTTCCCTCAACATCAAGGCTATGACCTTCGACGACACCATCGGCGTGCTGT
CTCAACGTGGTCCCGAGTTCGACAACCTGTCTGCTACCGCTGGTTCCCCTATCGTGCTGCAACGTCGTGGTTCCGTCTAC
GGTGCTAACGTGCCCATGATCACCGAGCTGGTCAACGACGGCAACGTCCAGTTCTTGGACCAGGACGACGACGATGACCC
CGACACCGAACTGTACCTGACTCAGCCTTTCGCTTGCGGCACCGCTTTCGCTGTGTCTGTCCTGGACTCCCTGATGTCCA
CCACCTACTTCAACCAGAATGCTCTGACCCTCATCCGTTCTCTGATCACCGGTGGCGCTACCCCTGAGCTGGAACTGATC
TTGGCTGAAGGCGCTGGACTGCGCGGTGGTTACTCTACTGTGGAATCCCTGTCCAACCGCGACCGTTGCCGTGTGGGTCA
AATCTCCTTGTACGACGGTCCCCTGGCTCAGTTCGGAGAGTGTGGCAAATACGGCGACCTGTTCGTGGCTGCTCTGAAGT
CCTACGGCATGCTGTGCATCGGCCTGTACCGTTTCCGTGACACCTCTTCCTCTTGCGACGCCTCCTCCAAGCGTTACGTC
ATCACTAACCCACCTGACGACTTCTCCCTGCTGCCTACCGACCAAGTGTTCGTGCTCATGCAGTTCGACCCCGGCCTCGA
GTACAAGCCTCCTGCTGTTAGGGCTCCAGCTGGTGGTCGTGGCACCAATACTCAAGGTTCCGGTGTCGGTGGTGGCGGTT
CCAACAAGGACGACAACTCCGGTTCCTCTGGAACCGAGACTTCCCAAGTGGCTCCCGCTTAA
```

The sequences corresponding to the GSSG linker, the Rho1D4 and the Stop codon are highlighted in cyan, yellow and red, respectively.

Supplementary Table 2. Cryo-EM data collection, refinement and validation statistics

	Ca^{2+}-bound Slo (EMDB-13700) (PDB 7PXE)	Ca^{2+}-free Slo (EMDB-13701) (PDB 7PXF)	Slo-Verruculogen (EMDB-13702) (PDB 7PXG)	Slo-Emodepside (EMDB-13703) (PDB 7PXH)
**Data collection and processing**				
Magnification	130,000			
Voltage (kV)	300			
Electron exposure (e–/Å^2)	74.0	79.8	80.4	75.4
Defocus range (μm)	-0.9 to -2.1			
Pixel size (Å)	0.35 (super-resolution) / 0.70 (native)			
Symmetry imposed	C4	C4	C4	C2
Initial particle images (no.)	906,619	920,897	1,131,410	1,861,584
Final particle images (no.)	225,754	90,833	66,077	261,505
Map resolution (Å)	2,38	2,68	2,73	2,59
FSC threshold	0.143	0.143	0.143	0.143
Map resolution range (Å)	2.0 – 3.1	2.4 – 3.8	2.4 – 4.0	2.2 – 3.4
**Refinement**				
Initial model used (PDB code)	Homology model based on PDB 5TJ6 (https://doi.org/10.2210/pdb5TJ6/pdb)			
Model resolution (Å)	2.5	2.9	2.9	2.7
FSC threshold	0.5	0.5	0.5	0.5
Map sharpening *B* factor (Å^2)	33.8	26.1	23.8	36.3
Model composition				
Non-hydrogen atoms	28,928	28,099	28,664	28,923
Protein residues	3,540	3,540	3,540	3,540
Ligands	700	7	572	829
Water	136	-	-	2
B factors (Å^2)				
Protein	83.8	102.8	74.2	82.4
Ligand	106.2	84.3	87.5	104.8
Water	67.8	-	-	83.2
R.m.s. deviations				
Bond lengths (Å)	0.002	0.002	0.002	0.002
Bond angles (°)	0.390	0.446	0.483	0.430
Validation				
MolProbity score	1.51	1.32	1.37	1.19
Clashscore	5.34	5.57	6.51	4.03
Poor rotamers (%)	1.94	0.65	1.03	0.26
Ramachandran plot				
Favored (%)	99.0	97.9	98.6	98.2
Allowed (%)	1.0	2.1	1.4	1.8
Disallowed (%)	0.0	0.0	0.0	0.0
