# Supplementary material for: Small molecule modulation of the Drosophila Slo channel elucidated by cryo-EM
Source: Nat Commun. 2021 Dec 9;12:7164. doi: 10.1038/s41467-021-27435-w (PMC8660915; doi:10.1038/s41467-021-27435-w)
Supplement: Supplementary file 3 — Description of Additional Supplementary Files [file 41467_2021_27435_MOESM3_ESM.pdf]

Description of Additional Supplementary Files

Supplementary Movie 1. Architecture and gating mechanism of *Drosophila* Slo

Slo comprises a transmembrane domain (TMD) and a domain-swapped intracellular gating ring composed of two RCK domains. The TMD consists of a selectivity filter embedded in a pore domain and a voltage sensor domain. Upon Ca^{2+}-binding, the RCK1 N-lobe moves outwards and pulls helix S6 of the TMD along. This leads to an expansion of the central cavity adjacent to the selectivity filter and activation of K^+ translocation.

Supplementary Movie 2. Predicted drug-binding pockets on the *Drosophila* Slo surface

Several potential binding pockets for small molecules were predicted in the gating ring and the transmembrane domain of Slo. These pockets offer the potential to design compounds binding to them that might lock Slo in either the open or the closed conformation.

Supplementary Movie 3. Verruculogen binding

Verruculogen binds to the previously identified S6 pocket and locks the transmembrane domain in the inactive conformation.

Supplementary Movie 4. Emodepside binding

Emodepside binds to the central cavity and serves as an additional ring below the selectivity filter.
