# Supplementary material for: Small molecule modulation of the Drosophila Slo channel elucidated by cryo-EM
Source: Nat Commun. 2021 Dec 9;12:7164. doi: 10.1038/s41467-021-27435-w (PMC8660915; doi:10.1038/s41467-021-27435-w)
Supplement: Supplementary file 8 — Reporting Summary [file 41467_2021_27435_MOESM8_ESM.pdf]

nature research

Corresponding author(s): Stefan Raunser

Last updated by author(s): Nov 16, 2021

Reporting Summary

Nature Research wishes to improve the reproducibility of the work that we publish. This form provides structure for consistency and transparency in reporting. For further information on Nature Research policies, see our Editorial Policies and the Editorial Policy Checklist.

Statistics

For all statistical analyses, confirm that the following items are present in the figure legend, table legend, main text, or Methods section.

n/a	Confirmed	
☐	☒	The exact sample size (*n*) for each experimental group/condition, given as a discrete number and unit of measurement
☒	☐	A statement on whether measurements were taken from distinct samples or whether the same sample was measured repeatedly
☒	☐	The statistical test(s) used AND whether they are one- or two-sided<br>*Only common tests should be described solely by name; describe more complex techniques in the Methods section.*
☒	☐	A description of all covariates tested
☒	☐	A description of any assumptions or corrections, such as tests of normality and adjustment for multiple comparisons
☐	☒	A full description of the statistical parameters including central tendency (e.g. means) or other basic estimates (e.g. regression coefficient) AND variation (e.g. standard deviation) or associated estimates of uncertainty (e.g. confidence intervals)
☒	☐	For null hypothesis testing, the test statistic (e.g. *F*, *t*, *r*) with confidence intervals, effect sizes, degrees of freedom and *P* value noted<br>*Give P values as exact values whenever suitable.*
☒	☐	For Bayesian analysis, information on the choice of priors and Markov chain Monte Carlo settings
☒	☐	For hierarchical and complex designs, identification of the appropriate level for tests and full reporting of outcomes
☒	☐	Estimates of effect sizes (e.g. Cohen's *d*, Pearson's *r*), indicating how they were calculated

Our web collection on statistics for biologists contains articles on many of the points above.

Software and code

Policy information about availability of computer code

Data collection	- Cryo-EM data: EPU version 2.7 and 2.8 (Thermo Fisher Scientific)<br>- Ephys data: Automated patch clamp (APC): Nanion PatchControl384PE 1.60, Manual patch clamp: PatchMaster v2x90.2
Data analysis	- Cryo-EM data: TranSPHIRE version 1.5.9, CTFFIND4 version 1.13, crYOLO version 1.7, WinCoot version 0.8.9, SPHIRE version 1.3, RELION version 3.1, PHENIX version 1.19, ChimeraX version 1.2.5<br>- Ephys Data: DataControl384 1.8.0; IGORPro6.34A, GraphPad Prism 8.02.

For manuscripts utilizing custom algorithms or software that are central to the research but not yet described in published literature, software must be made available to editors and reviewers. We strongly encourage code deposition in a community repository (e.g. GitHub). See the Nature Research guidelines for submitting code & software for further information.

Data

Policy information about availability of data

All manuscripts must include a data availability statement. This statement should provide the following information, where applicable:

- Accession codes, unique identifiers, or web links for publicly available datasets
- A list of figures that have associated raw data
- A description of any restrictions on data availability

The atomic coordinates and cryo-EM maps for Slo alone and in complex with verruculogen and emodepside are available at the Protein Data Bank (PDB)/Electron Microscopy Data Bank (EMDB) databases. The accession numbers are 7PXE (https://doi.org/10.2210/pdb7PXE/pdb) and EMD-13700 (https://wwwdev.ebi.ac.uk/emdb/EMD-13700) for Ca2+-bound apo, 7PXE (https://doi.org/10.2210/pdb7PXF/pdb) and EMD-13700 (https://wwwdev.ebi.ac.uk/emdb/EMD-13701) for Ca2+-free apo, 7PXE (https://doi.org/10.2210/pdb7PXF/pdb) and EMD-13700 (https://wwwdev.ebi.ac.uk/emdb/EMD-13702) for verruculogen, and 7PXE (https://doi.org/10.2210/pdb7PXH/pdb) and EMD-13700 (https://wwwdev.ebi.ac.uk/emdb/EMD-13703) for emodepside, respectively.

Other publicly available data that have been used and cited in this work: DNA sequnce of Slowpoke, isoform J from Drosphila (NCBI reference sequence NP_001014658.1; https://www.ncbi.nlm.nih.gov/protein/NP_001014658.1); structure of Aplysia Slo (PDB 5TJ6; https://doi.org/10.2210/pdb5TJ6/pdb), structure of KscA (PDB 1K4C; https://doi.org/10.2210/pdb1K4C/pdb).

Field-specific reporting

Please select the one below that is the best fit for your research. If you are not sure, read the appropriate sections before making your selection.

☒ Life sciences ☐ Behavioural & social sciences ☐ Ecological, evolutionary & environmental sciences

For a reference copy of the document with all sections, see nature.com/documents/nr-reporting-summary-flat.pdf

Life sciences study design

All studies must disclose on these points even when the disclosure is negative.

Sample size	Automated patch clamp experiments were performed in 24 replicates per concentration (one row of a 384 well plate). According to experience 24 replicates are sufficient to gain enough datapoints for reliable dose response curve calculations.
Data exclusions	A) automated selection by patch control software (quality parameter)<br>B) manual exclusion if controls were inappropriate (resulting in at least 3 up to 16 valid datapoints)
Replication	All 24 replicates per concentration were performed in one experiment on a 384-well plate
Randomization	The study does not contain experiments where randomization would be necessary. Covariances were excluded by treating samples in an automated, unbiased and independent way.
Blinding	The study does not contain experiments where blinding would be applicable. Experiments were conducted and controlled in an automated manner, thereby excluding potential bias by the experimenter.

Reporting for specific materials, systems and methods

We require information from authors about some types of materials, experimental systems and methods used in many studies. Here, indicate whether each material, system or method listed is relevant to your study. If you are not sure if a list item applies to your research, read the appropriate section before selecting a response.

Materials & experimental systems

n/a	Involved in the study	
☒	☐	Antibodies
☐	☒	Eukaryotic cell lines
☒	☐	Palaeontology and archaeology
☒	☐	Animals and other organisms
☒	☐	Human research participants
☒	☐	Clinical data
☒	☐	Dual use research of concern

Methods

n/a	Involved in the study	
☒	☐	ChIP-seq
☒	☐	Flow cytometry
☒	☐	MRI-based neuroimaging

Eukaryotic cell lines

Policy information about cell lines

Cell line source(s)	Purification: T.ni High Five cells were obtained from Thermo Fisher Scientific (cat. no. B85502).<br>Electrophysiology: the CHO cell line (ATCC CRL-9096) stably expressing Slo from Drosophila melanogaster (GenBank: AAA28651.1) was generated at Bayer Crop Science (for details see Crisford et al. 2015).
Authentication	Slo CHO cells: Slo channel function were assesed electrophysiologically, i.e. the measured currents of the control samples were compared to previously published results using similar experimental setups (see Crisford et al. 2015).<br>The T.ni cell line was not authenticated.
Mycoplasma contamination	The Slo CHO cell line was tested negative for mycoplasma contamination (regular checks every half year).<br>T.ni cells were not tested for macoplasma since they were not cultured for prolonged time periods.
Commonly misidentified lines (See ICLAC register)	None
